# The Impact of Coronary Artery Disease on Outcomes in Patients Hospitalized With Pre-eclampsia

**DOI:** 10.7759/cureus.59309

**Published:** 2024-04-29

**Authors:** Omar Elkattawy, Yash Shah, Shakhzoda Alimdjanova, Mina Ghbrial, Jahanzeb Javed, Omar Mohamed, Manik Dayal, Afif Hossain, Sherif Elkattawy, Fayez Shamoon

**Affiliations:** 1 Internal Medicine, Rutgers University New Jersey Medical School, Newark, USA; 2 Radiology, Rutgers University New Jersey Medical School, Newark, USA; 3 Medicine, Saint Barnabas Medical Center, Livingston, USA; 4 Cardiology, Saint Joseph University Medical Center, Paterson, USA

**Keywords:** preventive cardiology, cardio-obstetrics, coronary artery disease, pre-eclampsia, cardiology

## Abstract

Introduction

Pre-eclampsia leads to long-lasting cardiovascular effects in women in the postpartum period, but prevalence and in-hospital adverse events of coronary artery disease (CAD) in women with pre-eclampsia are poorly understood. The prevalence, outcomes, and mortality risks identified in this study allow for possible routes of clinical intervention of CAD in women with pre-eclampsia. The purpose of this study was to determine the prevalence and outcomes of CAD in women diagnosed with pre-eclampsia compared to those with pre-eclampsia with no history of CAD. Predictors of mortality in pre-eclampsia were also analyzed.

Methods

Data were obtained from the National Inpatient Sample from January 2016 to December 2019. We used the multivariate logistic regression to assess the independent association of CAD with outcomes in patients admitted with pre-eclampsia. We also used the multivariate logistic regression to analyze predictors of mortality in patients hospitalized with pre-eclampsia.

Results

Women with pre-eclampsia admitted between January 2016 and December 2019 were included in our analysis. A total of 256,010 patients were diagnosed with pre-eclampsia. Of these patients, 174 (0.1%) patients had CAD. Multivariate analysis demonstrated that CAD in patients with pre-eclampsia was independently associated with angioplasty (adjusted odds ratio [aOR] 62.28; 95% CI 20.459-189.591; p=0.001), permanent pacemaker (aOR 35.129; 95% CI 13.821-89.287; p=0.001), left heart catheterization (aOR 29.416; 95% CI 7.236-119.557; p=0.001), non-ST-elevation myocardial infarction (NSTEMI) (aOR 25.832; 95% CI 7.653-87.189; p=0.001), and congestive heart failure (CHF) (aOR 13.948; 95% CI 7.648-25.438; p=0.001). We also used the multivariate logistic regression model to assess predictors of mortality in patients admitted with pre-eclampsia. These included age at admission (aOR 1.064; 95% CI 1.009-1.121; p=0.021), Asian/Pacific-Islander race (aOR 4.893; 95% CI 1.884-12.711; p=0.001), and comorbidities such as CHF (aOR 19.405; 95% CI 6.408-58.768; p=0.001), eclampsia (aOR 17.253; 95% CI 5.323-55.924; p=0.001), syndrome of HELLP (hemolysis, elevated liver enzymes, low platelets) (aOR 6.204; 95% CI 2.849-13.510; p=0.001), coagulopathy (aOR 6.524; 95% CI 1.997-21.308; p=0.002), and liver disease (aOR 5.217; 95% CI 1.156-23.554; p=0.032).

Conclusion

In a large cohort of patients admitted with pre-eclampsia, we found the prevalence of CAD to be 0.1%. CAD was associated with several clinical outcomes, including NSTEMI. Predictors of mortality in patients with pre-eclampsia included demographic variables such as age and Asian race, as well as comorbidities such as CHF and coagulopathy.

## Introduction

Pre-eclampsia, a hypertensive disorder of pregnancy, is estimated to occur in one out of 25 pregnancies in the United States and is one of the leading causes of maternal morbidity and mortality [1]. The hallmarks of pre-eclampsia include new-onset hypertension after 20 weeks of gestation, accompanied by proteinuria or signs of organ system or uteroplacental dysfunction. While the pathogenesis of pre-eclampsia remains poorly understood, it is hypothesized that the resultant oxidative stress from placental hypoperfusion and hypoxia trigger a poorly moderated inflammatory response, causing systemic vasoconstriction and endothelial dysfunction, which ultimately result in systemic hypertension and end-organ hypoperfusion and dysfunction [2,3]. Although pre-eclampsia is confined to pregnancy with the only definitive cure being delivery, there is growing evidence that these effects on end organs persist after pregnancy [4]. Specifically, pre-eclampsia and other hypertensive disorders of pregnancy have been shown to increase the subsequent risk of cardiovascular disease in affected mothers.

Multiple studies have found that hypertension is more prevalent in women with pre-eclampsia after pregnancy [5-7], with 41.5% of women with a history of severe pre-eclampsia, in one study, diagnosed with hypertension within one year of delivery [7]. It has also been shown that a history of pre-eclampsia is an independent risk factor for coronary artery calcifications (CACs), with women aged 40-45 years developing CACs five years earlier and at a four-times greater likelihood of having CACs than their normotensive counterparts [8]. Although it has been well-established that pre-eclampsia predisposes women to a higher risk of cardiovascular disease, the impact of coronary artery disease (CAD) on outcomes within the cohort of women diagnosed with pre-eclampsia has not been studied in depth.

## Materials and methods

Data acquisition

This is a retrospective database study of the National Inpatient Sample (NIS) database. The NIS is part of the Healthcare Cost and Utilization Project (HCUP) set forth by the Agency for Healthcare Research and Quality. It uses the International Classification of Disease, Tenth Edition, Clinical Modification (ICD-10-CM) codes for diagnosis and procedures. The data set was used to examine patients admitted between the years 2016 and 2019. Encounters with primary diagnosis of pre-eclampsia were selected using ICD-10 code O14. This cohort of patients was further divided into patients with CAD using ICD-10 code I25.1 versus patients without CAD. Adult patients aged 18 years and older were included. We abstracted data from 260,055 charts, excluded 4,045, and were left with 256,010 charts for analysis. IRB approval was not required as the NIS database provides de-identified patient information.

Outcomes and variables

Patient baseline characteristics such as age, race, and insurance status were extracted. Comorbidities, hospital complications, mortality rates, disposition status, length of stay, and total charges were also analyzed.

The primary aim of the study was to assess whether or not there is a difference in outcomes (mortality, in-hospital complications, length of stay, total charges) between the cohort of patients with pre-eclampsia and CAD and patients with pre-eclampsia and without CAD. We also analyzed the independent association of CAD with outcomes after controlling for confounders such as age, race, and comorbidities. A secondary aim of the study was to evaluate predictors of mortality among patients hospitalized with pre-eclampsia.

Statistical analysis

Categorical values were analyzed using Pearson chi-square analysis, and continuous variables were analyzed using independent Student’s t-test. Logistic regression was performed to generate odds ratio with 95% confidence intervals (CIs) to assess predictors of mortality in patients with pre-eclampsia. We also used logistic regression to assess the independent association of CAD with clinical outcomes after controlling for confounders such as age, race, and comorbidities. A p-value of <0.05 was considered statistically significant. All analyses were completed using IBM SPSS Statistics for Windows, Version 29.0 (IBM Corp., Armonk, NY).

## Results

From January 2016 to December 2019, a total of 256,010 patients were diagnosed with pre-eclampsia. Of these patients, 174 (0.1%) patients had CAD. A statistical analysis of baseline characteristics is summarized in Table 1. Discharge disposition yielded a statistically significant result (p=0.001), underscoring differences in discharge disposition between patients with pre-eclampsia with and without an underlying CAD diagnosis. The variations observed were in the distribution of discharge dispositions, with CAD patients experiencing more frequent transfers to short-term hospitals (3 [1.7%] vs 2589 [1%]) and discharge with home health care (4 [2.3%] vs 5,418 [2.1%]). Subsequent analysis explored the impact of racial differences on the observed proportions of pre-eclampsia in patients with and without underlying CAD. CAD was more prevalent among Black race (62 [36.7%] vs 56,299 [22.8%]) and Asian or Pacific Islander race (10 [5.9%] vs 11,027 [4.5%]). The analysis revealed a statistically significant difference in the presence of diagnosed CAD in patients with pre-eclampsia based on race (p=0.001). T-test comparisons revealed CAD was associated with increased age at admission (32.89 vs 29.38, p=0.001), total healthcare expenses incurred during hospital stay ($53,694.06 vs $32,736.80, p=0.001), and length of hospital stay (6.46 days vs 4.13 days, p=0.001).

**Table 1:** Baseline characteristics of the study population of pre-eclampsia patients stratified according to with and without CAD Data are presented as n (%) CAD, coronary artery disease

Variable	No CAD	CAD	P-value
Disposition of patient	-	-	0.001
Routine	245,180 (95.9)	159 (91.4)	
Transfer to short-term hospital	2589 (1)	3 (1.7)	
Transfer other: includes skilled nursing facility, intermediate care facility, and another type of facility	386 (0.2)	0 (0)	
Home health care	5,418 (2.1)	4 (2.3)	
Against medical advice	2,155 (0.8)	8 (4.6)	
Died in hospital	44 (<0.1)	0 (0)	
Primary expected payer	-	-	0.137
Medicare	2,812 (1.1)	5 (2.9)	
Medicaid	113,999 (44.6)	86 (49.7)	
Private insurance	127,103 (49.7)	77 (44.5)	
Self-pay	5,169 (2)	3 (1.7)	
No charge	191 (0.1)	0 (0)	
Other	6281 (2.5)	2 (1.2)	
Race	-	-	0.001
White	116,582 (47.2)	67 (39.6)	
Black	56,299 (22.8)	62 (36.7)	
Hispanic	50,259 (20.4)	25 (14.8)	
Asian or Pacific Islander	11,027 (4.5)	10 (5.9)	
Native American	2,447 (1)	0 (0)	
Other	10,254 (4.2)	5 (3)	
Age in years at admission	29.38	32.89	0.001
Total charges during hospital stay (USD)	32,736.80	53,694.06	0.001
Length of hospital stay (days)	4.13	6.46	0.001

Univariate analysis was employed to assess the burden of various comorbidities in patients with and without CAD (Table 2). CAD patients had a higher burden of comorbidities including congestive heart failure (CHF) (33 [19%] vs 1,117 [0.4%], p=0.001), chronic obstructive pulmonary disease (31 [17.8%] vs 19,354 [7.6%], p=0.001), coagulopathy (4 [2.3%] vs 2,039 [0.8%], p=0.026), type 2 diabetes (37 [21.3%] vs 7,173 [2.8%], p=0.001), liver disease (3 [1.7%] vs 991 [0.4%], p=0.005), atrial fibrillation (3 [1.7%] vs 170 [0.1%], p=0.001), pulmonary hypertension (9 [5.2%] vs 488 [0.2%], p=0.001), obstructive sleep apnea (OSA) (10 [5.7%] vs 1,429 [0.6%], p=0.001), obesity (61 [35.1%] vs 49,568 [19.4%], p=0.001), and end-stage renal disease (ESRD) (1 [0.6%] vs 96 [<0.1%], p=0.001).

**Table 2:** Prevalence of comorbidities in the study population of pre-eclampsia patients with and without CAD Values are presented as n (%). AF, atrial fibrillation; AUD, alcohol use disorder; CAD, coronary artery disease; CHF, congestive heart failure; COPD, chronic obstructive pulmonary disease; CUD, cocaine use disorder; CVD, cerebrovascular disease; ESRD, end-stage renal disease; HIV, human immunodeficiency virus; HTN, hypertension; IDA, iron deficiency anemia; OSA, obstructive sleep apnea; OUD, opioid use disorder; PVD, peripheral vascular disease; PVD, peripheral vascular disease; T2DM, type 2 diabetes mellitus; TUD, tobacco use disorder

Variable	No CAD	CAD	P-value
IDA	6,629 (2.6)	7 (4)	0.235
CHF	1,117 (0.4)	33 (19)	0.001
COPD	19,354 (7.6)	31 (17.8)	0.001
Coagulopathy	2,039 (0.8)	4 (2.3)	0.026
CVD	343 (0.1)	0 (0)	0.629
T2DM	7,173 (2.8)	37 (21.3)	0.001
HTN	648 (0.3)	1 (0.6)	0.399
AUD	355 (0.1)	0 (0)	0.623
Liver disease	991 (0.4)	3 (1.7)	0.005
PVD	44 (<0.1)	0 (0)	0.863
AF	170 (0.1)	3 (1.7)	0.001
Hypothyroidism	11,820 (4.6)	10 (5.7)	0.479
HIV	76 (<0.1)	0 (0)	0.82
Pulmonary hypertension	488 (0.2)	9 (5.2)	0.001
TUD	207 (0.1)	0 (0)	0.707
OSA	1,429 (0.6)	10 (5.7)	0.001
Gestational DM	30,166 (11.8)	13 (7.5)	0.077
CUD	869 (0.3)	2 (1.1)	0.067
OUD	2,248 (0.9)	1 (0.6)	0.668
Obesity	49,568 (19.4)	61 (35.1)	0.001
ESRD	96 (<0.1)	1 (0.6)	0.001

The study also identified higher incidence of several clinical complications and outcomes within the CAD cohort (Table 3). Hospitalized patients with pre-eclampsia and concomitant CAD had higher rates of cardiac arrest (2(1.1%) vs 102(<0.1%), p=0.001), permanent pacemaker (ppm) implantation (48 [27.6%] vs 172 [<0.1%], p=0.001), ventricular fibrillation (2 [1.1%] vs 8 [<0.1%], p=0.001), and ventricular tachycardia (2 [1.1%] vs 90 [<0.1%], p=0.001). There were also higher rates of angioplasty (41 [23.6%] vs 20 [<0.1%], p=0.001), pulmonary embolism (1 [0.6%] vs 112 [<0.1%], p=0.001), left heart catheterization (LHC) (5 [2.9%] vs 23 [<0.1%], p=0.001), mechanical ventilation (3 [1.7%] vs 275 [0.1%], p=0.001), and non-ST-elevation myocardial infarction (NSTEMI) (8 [4.6%] vs 42 [<0.1%], p=0.001). Additionally, a significant difference was observed in the occurrence of shock following delivery (1 [0.6%] vs 253 [<0.1%], p=0.046) between the two patient cohorts.

**Table 3:** Outcomes of the study population of pre-eclampsia patients with and without CAD Values are presented as n (%) IABP, intra-aortic balloon pump; LHC, left heart catheterization; PPM, permanent pacemaker; RHC, right heart catheterization; VF, ventricular fibrillation; VT, ventricular tachycardia; NSTEMI, non-ST elevation myocardial infarction; STEMI, ST-segment elevation myocardial infarction

Variable	No CAD	CAD	P-value
In-hospital mortality	44 (<0.1)	0 (0)	0.863
Cardiac arrest	102 (<0.1)	2 (1.1)	0.001
PPM	172 (<0.1)	48 (27.6)	0.001
Cardiac tamponade	8 (<0.1)	0 (0)	0.941
VF	8 (<0.1)	2 (1.1)	0.001
VT	90 (<0.1)	2 (1.1)	0.001
Angioplasty	20 (<0.1)	41 (23.6)	0.001
Pulmonary embolism	112 (<0.1)	1 (0.6)	0.001
Eclampsia	746 (<0.1)	0 (0)	0.476
HELLP	10,982 (4.3)	5 (2.9)	0.356
Shock after delivery	253 (0.1)	1 (0.6)	0.046
Postpartum hemorrhage	18,655 (7.3)	8 (4.6)	0.172
RHC	25 (<0.1)	0 (0)	0.896
LHC	23 (<0.1)	5 (2.9)	0.001
Cardiogenic shock	29 (<0.1)	0 (0)	0.888
IABP	5 (<0.1)	0 (0)	0.953
Mechanical ventilation	275 (0.1)	3 (1.7)	0.001
Vasopressor use	348 (0.1)	1 (0.6)	0.117
STEMI	8 (<0.1)	0 (0)	0.941
NSTEMI	42 (<0.1)	8 (4.6)	0.001

Multivariate logistic regression analysis was employed to assess the independent association of CAD with outcomes in patients with pre-eclampsia, adjusting for possible confounders such as age, race, and comorbidities. This multivariable model shows CAD was independently associated with angioplasty (adjusted odds ratio [aOR] 62.28; 95% CI 20.459-189.591; p=0.001), permanent pacemaker placement (aOR 35.129; 95% CI 13.821-89.287; p=0.001), LHC (aOR 29.416; 95% CI 7.236-119.557; p=0.001), and NSTEMI (aOR 25.832; 95% CI 7.653-87.189; p=0.001).

**Figure 1:**
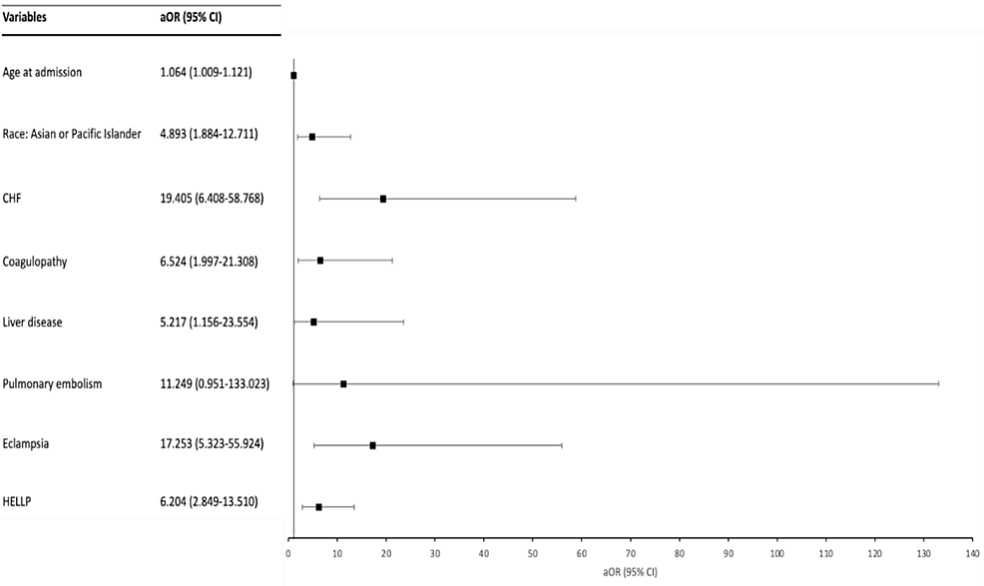
Predictors of mortality in patients admitted with pre-eclampsia aOR, adjusted odds ratio; CHF, congestive heart failure; CI, confidence interval

We also used the multivariate logistic regression model (Figure 1) to analyze the predictors of mortality in patients with pre-eclampsia. Age at admission (aOR 1.064; 95% CI 1.009-1.121; p=0.021), Asian/Pacific-Islander race (aOR 4.893; 95% CI 1.884-12.711; p=0.001), coagulopathy (aOR 6.524; 95% CI 1.997-21.308; p=0.002), liver disease (aOR 5.217; 95% CI 1.156-23.554; p=0.032), CHF (aOR 19.405; 95% CI 6.408-58.768; p=0.001), eclampsia (aOR 17.253; 95% CI 5.323-55.924; p=0.001), and syndrome of HELLP (hemolysis, elevated liver enzymes, low platelets) (aOR 6.204; 95% CI 2.849-13.510; p=0.001) were found to be predictors of mortality. Pulmonary embolism approached clinical significance for increased mortality risk but was not statistically significant (aOR 11.249; 95% CI 0.951-133.023, p=0.055).

## Discussion

In this study looking at the prevalence of CAD in patients admitted with pre-eclampsia from 2016 to 2019, we found the prevalence to be 0.1%. First, CAD was found to be more prevalent among African-American and Asian or Pacific Islander races. In the multivariate analysis looking at independent association of CAD with outcomes, we found CAD to be associated with LHC, angioplasty, permanent pacemaker placement, and NSTEMI. Predictors of mortality in patients with pre-eclampsia included age at admission, Asian or Pacific Islander race, CHF, liver disease, coagulopathy, eclampsia, and syndrome of HELLP.

The higher prevalence of CAD within African-American population can be explained by a higher prevalence of hypertension and higher burden of cardiovascular disease in African-American patients, which may be compounded by pre-eclampsia in pregnancy [9]. However, our logistic regression analysis showed no statistically significant differences in increased mortality risk for African-American patients with pre-eclampsia. Interestingly, logistic regression showed a significant mortality risk in Asian/Pacific-Islander women with pre-eclampsia, with a 4.9 times greater likelihood of mortality as an Asian/Pacific-Islander versus White women with pre-eclampsia. These findings are supported by another study focused on racial disparities in women with pregnancy-induced hypertension. The study found that while African-American women had the highest risk of experiencing pre-eclampsia, they had the lowest risk of cardiovascular complications after having pre-eclampsia/eclampsia. The highest risk of complications, including peripartum cardiomyopathy, heart failure, and ischemic heart disease, occurred in Asian/Pacific Islander women with pre-eclampsia or eclampsia [10]. This highlights the need for interventions in postpartum cardiovascular risk assessment in women of all races, with further consideration given to Asian/Pacific-Islander women.

The association of various comorbidities among women with pre-eclampsia and CAD was statistically significant when considering CHF, atrial fibrillation, coagulopathy, COPD, pulmonary hypertension, liver disease, type 2 diabetes, obstructive sleep apnea, and end-stage renal disease, as compared to those without a diagnosis of CAD. Some of these comorbidities, including obesity, type 2 diabetes, and chronic renal disease, are established independent risk factors for CAD [11]. The multisystem involvement of these conditions provides an opportunity for comprehensive medical optimization of risk factors to mitigate the development and complications of CAD in women with a history of pre-eclampsia.

During pregnancy, pre-eclampsia is associated with cardiovascular changes including diastolic dysfunction and left ventricular remodeling, which may persist and worsen after delivery [12]. These changes, along with endothelial dysfunction, are associated with the development of heart failure with preserved ejection fraction [5]. Our logistic regression looking at predictors of mortality in patients with pre-eclampsia found that CHF was the most significant predictor of mortality, with women with pre-eclampsia and CHF having a 19.4 times greater risk of mortality than those without CHF. These findings are consistent with the complications of pre-eclampsia, as a population-based study in Taiwan found that there is a persistently high incidence of myocardial infarction, heart failure, and stroke in women with pre-eclampsia up to three years postpartum, suggesting close monitoring of women with pre-eclampsia for years after delivery [13].

The two-way link between CAD and hypertensive disorders of pregnancy has long been a topic of research, even though the exact pathophysiology remains unclear. For one, CAD and pre-eclampsia share many common risk factors, including alcohol intake, smoking, and obesity [14,15]. Additionally, an underlying mechanism through endothelial dysfunction may be the common link between pre-eclampsia and future cardiovascular events [16]. Furthermore, some studies have shown that women with a family history of atherosclerotic disease are at an increased risk of pregnancy complications including pre-eclampsia and pregnancy losses, and that, conversely, pre-eclampsia may induce a greater risk of atherosclerotic disease [17].

While the association of pre-eclampsia with an increased risk of future cardiovascular complications has been established, the inverse relationship is not well understood. Studies have shown that pre-existing cardiovascular risk factors prior to pregnancy play a role in the pathogenesis of pre-eclampsia, including poor cardiovascular reserve [18]. In a study focused on examining the lipid profile of women with pre-eclampsia, it was found that higher levels of triglycerides, ApoE, and ApoB/ApoA1 were associated with a higher risk of pre-eclampsia [19]. Other studies have reported that dyslipidemia is a significant risk factor for developing pre-eclampsia, implicating excess triglyceride deposition in uterine spiral arteries as a contributor to endothelial dysfunction [20,21]. Common cardiovascular risk factors for CAD and pre-eclampsia, such as dyslipidemia, obscure causative relationships between these two conditions. Regardless, our study provides an opportunity for clinicians to hone in on preventative strategies for CAD in women with a history of pre-eclampsia.

Our study has the following limitations. NIS is an administrative database that uses ICD-10 codes, thus prone to human errors in coding. Confounders such as medication use are not provided by the NIS database and are, therefore, not accounted for in our study. Given the nature of the NIS database, it is unclear how the diagnosis of CAD was made, i.e., stress test vs LHC; instead, we relied on ICD-10 code to select patients with CAD. Lastly, long-term outcomes in patients cannot be established because patients were not followed longitudinally.

## Conclusions

In this study examining the association of CAD with clinical outcomes among patients hospitalized with pre-eclampsia, we arrived at several key findings. The analysis of clinical complications and outcomes reaffirmed the heightened risks faced by women with pre-eclampsia and CAD, including a higher incidence of cardiac arrest, permanent pacemaker implantation, ventricular fibrillation, ventricular tachycardia, angioplasty, pulmonary embolism, LHC, mechanical ventilation, and NSTEMI. Notably, the occurrence of shock following delivery demonstrated a significant difference between the two patient cohorts, underscoring the need for heightened vigilance and tailored interventions in managing postpartum complications. Our second multivariate logistic regression model evaluating predictors of mortality among all patients hospitalized with pre-eclampsia found that demographic variables, such as age and race, and comorbidities, such as CHF, coagulopathy, and liver disease, were all significant predictors of mortality. In conclusion, the findings of this study emphasize the need for comprehensive postpartum care beyond the gestational period, including continued monitoring and proactive management in the postpartum period. The insights gained from this study have implications for clinical practice, guiding healthcare professionals in the development of tailored interventions to mitigate the heightened cardiovascular risks faced by women with pre-eclampsia.
